# Jun Dimerization Protein 2 Activates *Mc2r* Transcriptional Activity: Role of Phosphorylation and SUMOylation

**DOI:** 10.3390/ijms18020304

**Published:** 2017-01-31

**Authors:** Chiung-Min Wang, Raymond X. Wang, Runhua Liu, Wei-Hsiung Yang

**Affiliations:** 1 Department of Biomedical Sciences, Mercer University School of Medicine, Savannah, GA 31404, USA; meowy200@yahoo.com (C.-M.W.); xw98@cornell.edu (R.X.W.); 2 Department of Genetics and Comprehensive Cancer Center, University of Alabama at Birmingham, Birmingham, AL 35294, USA; runhua@uab.edu

**Keywords:** Jun dimerization protein 2 (JDP2), melanocortin 2 receptor (MC2R), transcriptional activity, phosphorylation, SUMOylation

## Abstract

Jun dimerization protein 2 (JDP2), a basic leucine zipper transcription factor, is involved in numerous biological and cellular processes such as cancer development and regulation, cell-cycle regulation, skeletal muscle and osteoclast differentiation, progesterone receptor signaling, and antibacterial immunity. Though JDP2 is widely expressed in mammalian tissues, its function in gonads and adrenals (such as regulation of steroidogenesis and adrenal development) is largely unknown. Herein, we find that JDP2 mRNA and proteins are expressed in mouse adrenal gland tissues. Moreover, overexpression of JDP2 in Y1 mouse adrenocortical cancer cells increases the level of melanocortin 2 receptor (MC2R) protein. Notably, *Mc2r* promoter activity is activated by JDP2 in a dose-dependent manner. Next, by mapping the *Mc2r* promoter, we show that cAMP response elements (between −1320 and −720-bp) are mainly required for *Mc2r* activation by JDP2 and demonstrate that −830-bp is the major JDP2 binding site by real-time chromatin immunoprecipitation (ChIP) analysis. Mutations of cAMP response elements on *Mc2r* promoter disrupts JDP2 effect. Furthermore, we demonstrate that removal of phosphorylation of JDP2 results in attenuated transcriptional activity of *Mc2r*. Finally, we show that JDP2 is a candidate for SUMOylation and SUMOylation affects JDP2-mediated *Mc2r* transcriptional activity. Taken together, JDP2 acts as a novel transcriptional activator of the mouse *Mc2r* gene, suggesting that JDP2 may have physiological functions as a novel player in MC2R-mediated steroidogenesis as well as cell signaling in adrenal glands.

## 1. Introduction

Transcription factor activator protein 1 (AP-1) has been known to regulate gene expression in response to a variety of pathological and physiological stimuli, such as growth factors, infections, and stresses. Therefore, AP-1 plays an important role in controlling cellular processes including apoptosis, differentiation, and proliferation [1]. AP-1 is a heterodimeric protein and mainly consists of proteins belonging to the c-Fos, c-Jun, activating transcription factor (ATF), and Jun dimerization protein (JDP) families. While c-Fos and c-Jun usually activate gene expression, Jun Dimerization protein 2 (JDP2) represses AP-1-mediated *trans*-activation by recruiting histone deacetylase 3 (HDAC3) to the promoter region [2,3]. In addition to AP-1 site, JDP2 also binds to cAMP responsive element (CRE) site in numerous *cis*-elements of the target genes [4]. Recent studies have demonstrated that JDP2 has not only DNA-binding activity but also histone-binding activity [5]. JDP2 also has the nucleosome assembly activity and the activity of inhibition of histone methyltransferase and histone acetyltransferase [6]. Overall, JDP2 has been shown to be involved in biological and cellular functions such as suppressing adipocyte differentiation [7], controlling replicative senescence [8], promoting skeletal muscle differentiation [9], mediating osteoclast differentiation [10], mediating atrial dilatation and atrial fibrillation [11], interacting with and regulating progesterone receptor [12,13], maintaining Epstein-Barr virus latency [14], controlling bone homeostasis and antibacterial immunity [15], and contributing to metastatic spread [16].

JDP2 also controls the expression of cell-cycle regulators such as cyclin A2, cyclin E2, and p16 to induce cell-cycle arrest via RB-p16 and p53-p21 pathways [8,17]. Several studies have shown that JDP2 is also involved in cancer development and progression. For example, in a severe combined immune-deficient mice study, JDP2 inhibited cell transformation and acted as a tumor suppressor [18]. Down-regulation of JDP2 is associated with tumor metastasis and poor prognosis in patients with pancreatic carcinoma [19]. However, *JDP2* gene activity is elevated in head and neck squamous cell carcinoma cell lines of tongue and larynx [20]. JDP2 has also been shown to potentiate development of hepatocellular carcinoma in a mice study [21]. Overall, these results suggest that JDP2 has a wide range of functional roles in biological processes and cancer development and progression, but the underlying mechanism is still poorly understood.

Melanocortin 2 receptor (MC2R), a G-protein couple receptor, is an important membrane receptor selectively involved in the adrenocorticotropic hormone (ACTH)-mediated signaling, which has been shown to be the major endocrine signaling pathway in adrenal cortex. The binding of ACTH to MC2R results in the activation of MAPK- and PKA-dependent signaling cascades [22]. Mutations in MC2R have been associated with familial glucocorticoid deficiency [22]. Though JDP2 is broadly expressed in the majority of the tissues in humans and animals (and the RNA expression of JDP2 was reported in HPA and GTEx datasets), the functional role of JDP2 in adrenal cortex is largely unknown. Since the ACTH-MC2R signaling pathway is the most important endocrine event in adrenal cortex, we assessed the function of JDP2 in *Mc2r* transcriptional activity in the present study. We investigated the expression levels of JDP2 in mouse adrenal glands as well as whether JDP2 is a novel activator of *Mc2r* transcription in multiple human and mouse cell lines. The majority of the transcription factors are functionally regulated by post-translational modifications (PTMs) such as phosphorylation, methylation, ubiquitination, and SUMOylation. JDP2 has been shown to be phosphorylated at T148 and phosphorylation of JDP2 by the c-Jun N-terminal kinase targets it for proteosomal degradation [23]. Another PTM, SUMOylation (conjugation of SUMO protein to the target substrates) has profound effects on regulating normal cell physiology, development, and tumorigenesis [24,25,26,27,28,29,30,31]. The manipulation of small ubiquitin-like modifier (SUMO) modification and processes has gained focus as a potential therapeutic intervention. Thus, in the present study we also conducted experiments to determine whether post-translational modifications (phosphorylation and SUMOylation) play a role in the transcriptional activity of JDP2.

## 2. Results

### 2.1. JDP2 Is Expressed in Adrenal Glands and Increases MC2R Level

**Figure 1:**
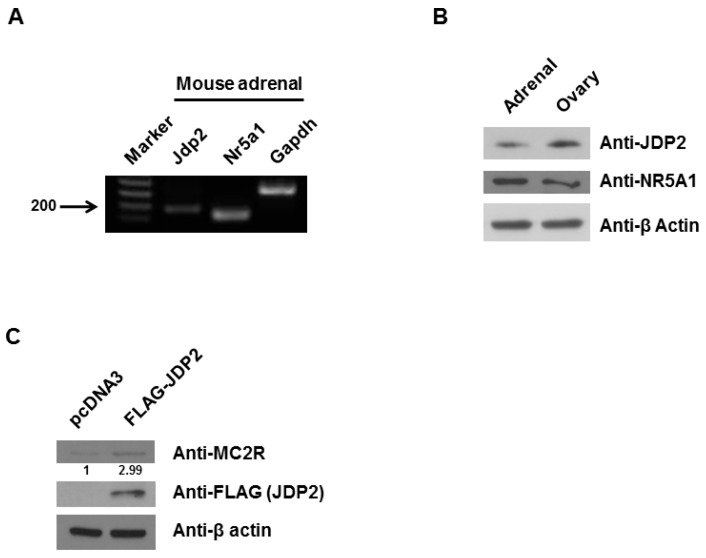
JDP2 is expressed in adrenal glands and increases melanocortin 2 receptor (MC2R) level. (**A**) RT-PCR analysis of *Jdp2* and *Nr5a1* expression from mouse adrenal glands. Total RNA was extracted and reverse transcribed to cDNA followed by PCR analysis and fractionation using agarose gel electrophoresis. The PCR product of *Jdp2* (189-bp) and the PCR product of *Nr5a1* (147-bp) are present in the adrenal glands. The PCR product of Glyceraldehyde-3-phosphate dehydrogenase (*Gapdh*) served as a control to indicate the presence of cDNA in each sample; (**B**) Western blot analysis of JDP2 and NR5A1 expression from mouse adrenal glands and ovaries. The expression levels of JDP2 and NR5A1 in adrenals and ovaries were determined using anti-JDP2 and anti-NR5A1 immunoblotting, respectively. The β-Actin levels were also determined for equal loading; (**C**) Y1 cells were transfected without or with FLAG-*Jdp2* expression plasmid and cell lysates were immunoblotted with anti-MC2R. The cells lysates were probed with a FLAG antibody (for JDP2 level) and a β-Actin antibody for equal loading, respectively. The experiments were performed three times.

The mRNA and protein levels of JDP2 in the adrenal glands of the mouse were determined using RT-PCR and western blot, respectively. Total RNA was extracted from the adrenal glands of adult mice. As shown in Figure 1A, RT-PCR revealed *Jdp2* transcripts in the adrenal glands. As expected, adrenal glands expressed transcripts of *Nr5a1* and *Gapdh* (housekeeping gene) as positive control. As shown in Figure 1B, both adrenal glands and ovaries expressed JDP2 proteins as NR5A1 protein levels as positive control. To determine whether JDP2 affects MC2R protein in Y1 adrenocortical cancer cells, expression vectors encoding wild-type *Jdp2* or empty vectors were transfected into Y1 cells. As shown in Figure 1C, when wild-type *Jdp2* was transfected, the level of MC2R protein was increased (approximately three-fold). These results indicate that adrenal glands express JDP2 protein and JDP2 enhances MC2R expression in Y1 adrenocortical cancer cells.

### 2.2. JDP2 Is an Activator of the Mc2r Promoter

**Figure 2:**
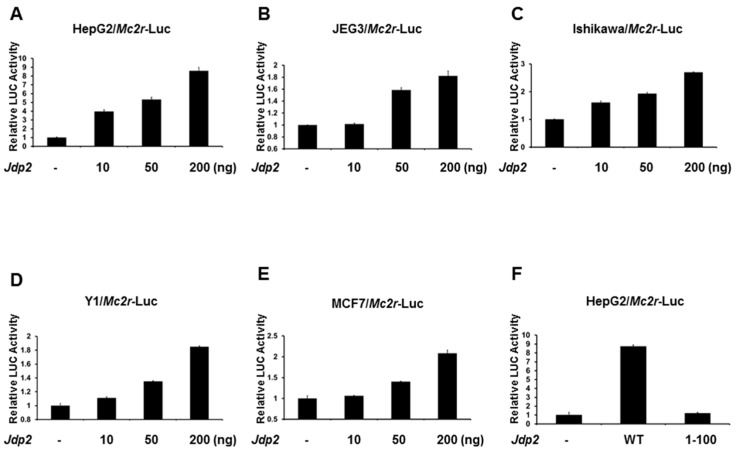
JDP2 activates *Mc2r* transcription. HepG2 (**A**), JEG3 (**B**), Ishikawa (**C**), Y1 (**D**), and MCF7 (**E**) cells were co-transfected with *Mc2r*-Luc and different amount of *Jdp2* plasmid; (**F**) HepG2 cells were co-transfected with *Mc2r*-Luc and either wild-type or 1-100 (loss of DNA binding domain) of *Jdp2* plasmid. Forty-eight hours after transfection, luciferase activities (LUC) were measured and normalized with Renilla activity. Relative LUC activity (presented as fold activation) was calculated and plotted on a graph. The experiments were conducted three times in triplicate setting. Error bars indicate the standard error. WT: Wild-type.

Since JDP2 is expressed in adrenal glands, we next determined whether the *Mc2r* promoter is regulated by JDP2. The −2000-bp *Mc2r* promoter-LUC reporter plasmid was cotransfected with wild-type *Jdp2* into several different cell lines and *Mc2r* promoter activity was determined by measuring LUC activity in cell lysates 48 h after transfection. As shown in Figure 2A–E, JDP2 dose-dependently activated *Mc2r* gene transcription in a LUC assay using HEPG2, JEG3, Ishikawa, Y1, and MCF7 cells. In order to prove the DNA binding domain is required for JDP2-mediated *Mc2r* promoter activity, we truncated out the DNA binding domain from the wild-type JDP2. As shown in Figure 2F, deletion of the DNA binding domain of JDP2 (1-100) was not able to activate *Mc2r* promoter. A similar result was observed in JEG3 cells (data not shown). These findings indicate that JDP2 is an activator of the *Mc2r* transcription.

### 2.3. Minimal Mc2r Promoter Region Responsive to JDP2 Activation

**Figure 3:**
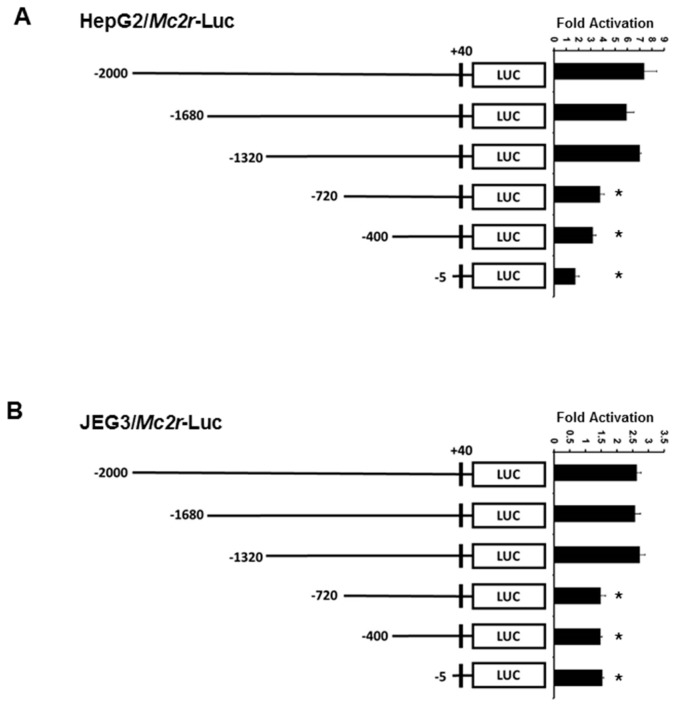
Regions of *Mc2r* promoter important for transcriptional activation by JDP2. HepG2 (**A**) and JEG3 (**B**) cells were co-transfected with mouse *Mc2r* promoter deletion constructs and *Jdp2* expression plasmid. Luciferase activities were measured 48 h after transfection and normalized with Renilla activity. Relative LUC activity (fold activation) was calculated and plotted. The experiments were performed three times in triplicate. Error bars indicate the standard error. * indicates two-tailed *t* test *p* < 0.05 vs. −2000-bp construct.

Because the −2000-bp mouse *Mc2r* promoter contains numerous cAMP response elements, the *Mc2r* promoter was truncated to determine the minimal region that is important for transcriptional activation by JDP2. As shown in Figure 3, three promoters of different lengths (−2000, −1680, −1320 bp) showed similar transcriptional activity by JDP2. Deletion of the promoter region which contains distal cAMP response elements, as shown in the −720 promoter, resulted in a significant loss (approximately 50%) of JDP2-mediated transcriptional activity in both HepG2 and JEG3 cells. This indicates that the distal cAMP response elements (between −1320 and −720 bp) are more important for JDP2 action on *Mc2r* promoter.

**Figure 4:**
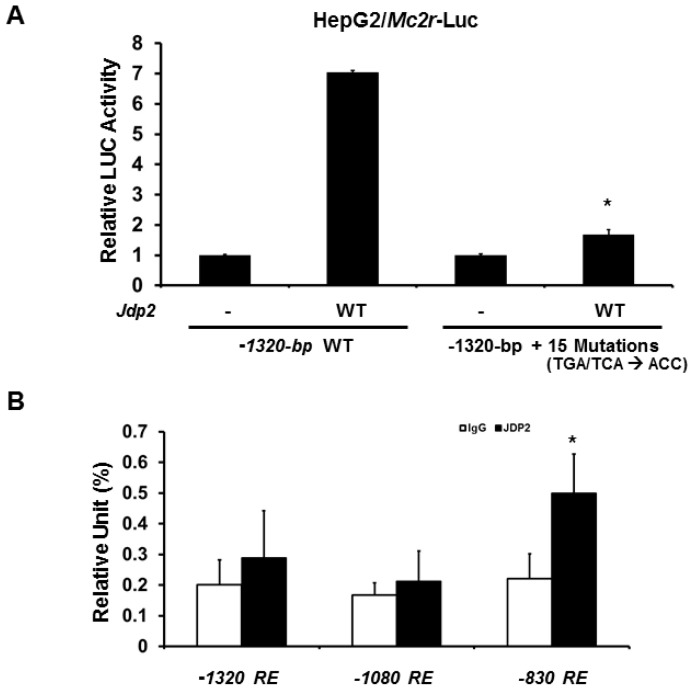
Mutations of cAMP response elements significantly reduce JDP2-mediated *Mc2r* promoter activity. (**A**) HepG2 cells were co-transfected *Jdp2* expression plasmid and with either wild-type or cAMP response element mutated *Mc2r* promoter −1320-bp constructs. Forty-eight hours after transfection, luciferase activities (LUC) were measured and normalized with Renilla activity. Relative LUC activity (presented as fold activation) was calculated and plotted on a graph. The experiments were performed three times in triplicate setting. Error bars indicate the standard error. * indicates two-tailed *t* test *p* < 0.05 vs. wild-type −1320-bp construct; (**B**) Quantification of the amounts of DNA precipitated, expressed as a percentage of the total input DNA, in ChIP analysis of JDP2-binding sites in the promoter region (between −3120 and −720-bp) of *Mc2r* in Y1 cells. The experiments were performed three times. * indicates two-tailed *t* test *p* < 0.05 vs. IgG. RE: response element.

To further determine whether the cAMP response elements are required for JDP2-mediated activation, we next generated 15 cAMP response element mutations (TGA or TCA were mutated to ACC) in −1320-bp *Mc2r* promoter-LUC plasmids. Consistent with the previous results, mutations of 15 cAMP response elements dramatically reduced (approximately 90% loss) JDP-mediated *Mc2r* promoter activity in HepG2 cells (Figure 4A). Similar results were observed in JEG3 cells (data not shown). To confirm JDP2 binds to *Mc2r* promoter region and because the promoter region between−1320 and −720-bp is the most important region for JDP2 binding, we next performed immunoglobin G (IgG) or JDP2 chromatin immunoprecipitation (ChIP) assay with qPCR analysis. As shown in Figure 4B, JDP2 binds to −830 cAMP response element predominately. Thus, cAMP response elements are indeed required for JDP-mediated transcriptional activation.

### 2.4. Phosphorylation of JDP2 at T148 Is Required for Full JDP2-Mediated Mc2r Transcriptional Activity

**Figure 5:**
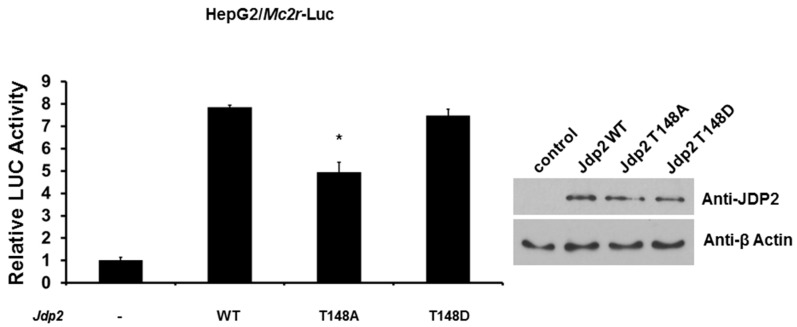
Loss of JDP2 phosphorylation reduces the activation of *Mc2r* promoter. HepG2 cells were co-transfected with a reporter plasmid with *Mc2r* promoter and either wild-type, T148A, or T148D *Jdp2* expression plasmid. Forty-eight hours after transfection, luciferase activities (LUC) were measured and normalized with Renilla activity. Relative LUC activity (presented as fold activation) was calculated and plotted on a graph. The protein levels of JDP2 in HepG2 cells from the reporter assays were confirmed using anti-JDP2 immunoblotting. The reporter assays were performed three times with similar results. * indicates two-tailed *t* test *p* < 0.05 vs. WT.

Because JDP2 has been shown to be phosphorylated at T148 by JNK (c-Jun N-terminal kinase) and p38 kinase [23], we next examined the effect of phosphorylation of JDP2 on transcriptional activity of *Mc2r* promoter. We cotransfected *Mc2r*-LUC reporter gene with either wild-type, T148A (mimicking de-phosphorylated), or T148D (mimicking phosphorylated) *Jdp2* expression plasmid and determined LUC activity 48 h post-transfection. As shown in Figure 5, while wild-type and T148D JDP2 enhanced *Mc2r* promoter activity eight-fold, T148A JDP2 significantly reduced this effect (approximately 38% loss). This result suggests that phosphorylation of JDP2 at T148 is required for full JDP2-mediated *Mc2r* promoter activity.

### 2.5. SUMOylation of JDP2 Affects Its Transcriptional Activities

**Figure 6:**
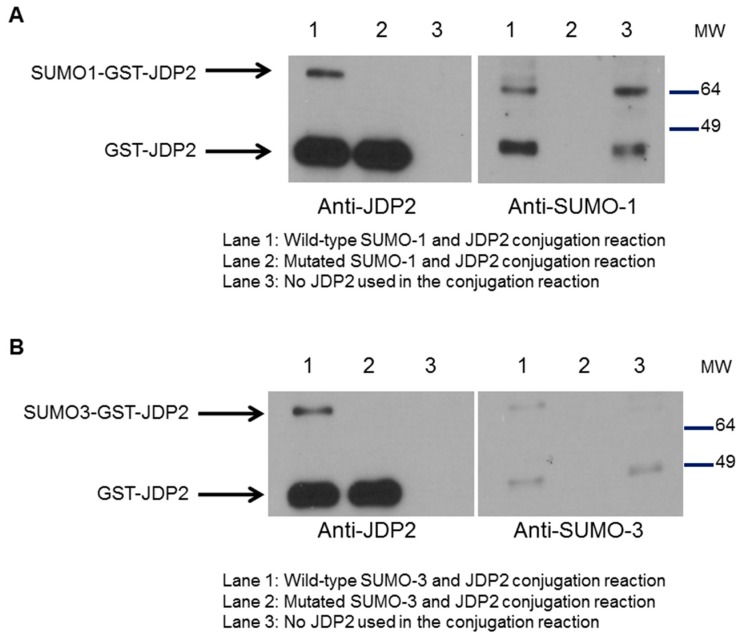
JDP2 is a novel substrate for SUMOylation. (**A**) In vitro SUMOylation of JDP2 with SUMO-1 wild-type and mutated form. GST-JDP2 protein was incubated with E1 and E2 enzymes and either wild-type or mutated SUMO-1 at 30 °C for 3 h. Western blot analysis was performed by immunoblotting with JDP2 antibody (**left**) or SUMO-1 antibody (**right**); (**B**) In vitro SUMOylation of JDP2 with SUMO-3 wild-type and mutated form. GST-JDP2 protein was incubated with E1 and E2 enzymes and either wild-type or mutated SUMO-3 at 30 °C for 3 h. Western blot analysis was performed by immunoblotting with JDP2 antibody (**left**) or SUMO-3 antibody (**right**). Experiments were performed three times with similar results. SUMO, small ubiquitin-like modifier. MW: molecular weight.

Previously, we have shown that ATF3 can be SUMOylated and SUMOylation/deSUMOylation of ATF3 regulates *TP53* gene activity and *trans*-activation of p53 [26]. Since JDP2 shares high homology with ATF3, we next investigated whether JDP2 is also modified by SUMO-1 and SUMO-3. GST-JDP2 protein was incubated with E1 and E2 enzymes and either wild-type or mutated SUMO-1 at 30 °C for 3 h followed by western blot analysis. As shown in Figure 6A, in vitro SUMOylation study reveals that JDP2 is capable of being modified by SUMO-1. A similar result was observed when SUMO-3 was used (Figure 6B). These results suggest that JDP2 is a novel substrate for SUMOylation.

**Figure 7:**
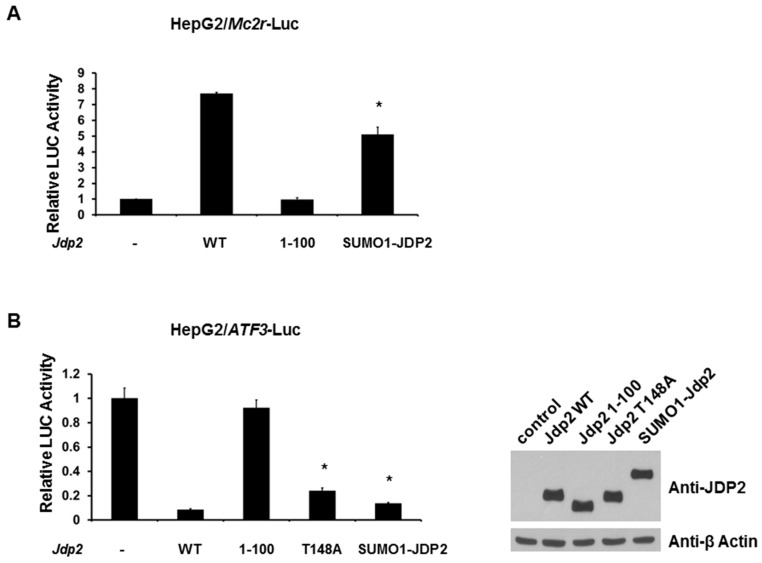
SUMOylation alters JDP2-mediated transcriptional activities. (**A**) HepG2 cells were co-transfected with a reporter plasmid with *Mc2r* promoter and either wild-type, 1-100, or SUMO1-fusion *Jdp2* expression plasmid; (**B**) HepG2 cells were co-transfected with a reporter plasmid with *ATF3* promoter and either wild-type, 1-100, T148A, or SUMO1-fusion *Jdp2* expression plasmid. Luciferase activities were measured 48 h after transfection and normalized with Renilla activity. Relative LUC activity (fold activation) was calculated and plotted. The expression levels of JDP2 in HepG2 cells from (**B**) were validated using anti-JDP2 immunoblotting. Experiments were performed three times with similar results. * indicates two-tailed *t* test *p* < 0.05 vs. WT.

Therefore, we next tested whether SUMOylation of JDP2 alters *Mc2r* promoter activities. As shown in Figure 7A, expression of wild-type JDP2 leads to an increase (seven to eight fold) in the activity of *Mc2r* promoter-driven LUC reporters. Notably, expression of the SUMO1-JDP2 (mimicking SUMOylated JDP2) reduced the activation by 30%. As expected, 1-100 JDP2 mutant (loss of DNA binding domain) was not able to activate *Mc2r* promoter.

Though JDP2 binds to ATF proteins, JDP2 suppresses the transcription of its homologue immediate early gene counterpart, ATF3 [32]. Because *ATF3* gene is one of the JDP2 target genes, we next assessed the SUMOylation effect of JDP2 on *ATF3* promoter. As shown in Figure 7B, while wild-type JDP2 significantly suppressed *ATF3* promoter, 1-100 JDP2 mutant (loss of DNA binding domain) was not able to repress *ATF3* promoter. Interestingly, we observed that both expression of T148A mutant protein and SUMO1-JDP2 fusion protein relieved the reduction by 20% and 10%, respectively. These results suggest that both phosphorylation and SUMOylation play a significant role in JDP2-mediated transcriptional activities.

## 3. Discussion

Transcription factors regulate target genes by responding to a wide variety of physiological stimuli and thus function as important mediators of development, metabolism, and reproduction. JDP2 is a transcription factor and widely expressed in tissues of humans and animals. However, its physiologic functions in the endocrine system, especially adrenal glands, is unknown. Herein, we show for the first time that JDP2 acts as a transcriptional activator of the *Mc2r* gene, including adrenocortical cancer cells.

Importantly, our data showed that JDP2 (mRNA and proteins) are normally expressed in mouse adrenal glands, indicating a possible role of JDP2 in adrenal gland development and steroid biosynthesis. Indeed, we observed that overexpression of JDP2 enhances the level of MC2R, a critical and essential receptor for ACTH function, in mouse Y1 adrenocortical cancer cells. Our promoter and ChIP analysis further supports the previous findings [4] that JDP2 binds to cAMP response elements in promoter regions to regulate target genes. Moreover, in agreement with other reports [5], we demonstrated that the DNA binding domain of JDP2 is required for JDP2-mediated *Mc2r* promoter activation.

In the present study, our data revealed that JDP2 is expressed in adrenal glands and up-regulates *Mc2r* promoter activity. NR5A1 has long been considered the major transcription factor in regulating MC2R activity [33]. The NR5A1 binding sites on human *MC2R* or mouse *Mc2r* promoters have been mapped. Previously, we have showed that FOXL2, an important transcription factor in ovarian development, synergistically activates *Mc2r* promoter with NR5A1 [34]. This information suggests that NR5A1, FOXL2, and JDP2 may coordinate together in regulating *Mc2r* transcriptional activity. One piece of data supporting our hypothesis is that our preliminary data suggests that JDP2 can interact with NR5A1 and regulates NR5A1-mediated transcription (unpublished data). Therefore, more studies are required to dissect how NR5A1, FOXL2, and JDP2 work together in regulating human *MC2R* and mouse *Mc2r* transcriptional activities and MC2R-mediated signaling pathways.

Reversible post-translational modifications (such as phosphorylation) which regulate protein functions in cells are one of the core principles in biochemistry and cell biology. As a transcription factor, JDP2, has been shown to be phosphorylated at T148 and this phosphorylation event is critical for JDP2’s turnover/degradation [23]. In the present work, we demonstrate that loss of phosphorylation on JDP2 T148 significantly reduces its activity in regulating *Mc2r* promoter. This further highlights that T148 is a critical PTM site for JDP2. In the past two decades another PTM, SUMOylation, has emerged as an important event in a variety of biological processes and regulations, including apoptosis, cell cycle regulation, genomic instability, inflammation, metabolism, transcriptional regulation, and tumor initiation and progression [24,25,26,27,28,29,30,31]. Previously, we have reported that ATF3 is a novel substrate for SUMOylation and that SUMOylation of ATF3 alters p53 *trans*-activation and stability as well as prostate cancer proliferation [26,35]. Since JDP2 exhibits 60% overall homology with ATF3 and the bZIP domains of these two proteins share 90% homology, we hypothesized that JDP2 can be SUMOylated by SUMO proteins. Indeed, our present study supports our hypothesis that JDP2 is a novel target protein for SUMOylation and that SUMOylation status of JDP2 alters its transcriptional activities of target genes. The reason why T48A mutation or SUMO-JDP2 fusion constructs from the present study still retain the ability to suppress ATF3 activation with albeit weaker reduction may be due to the strong interaction of JDP2-HDACs. A previous study [3] has shown that both JDP2 and ATF3 bind strongly to several HDACs, resulting in significant promoter suppression, such as the suppression of ATF3 promoter. Therefore, phosphorylation and SUMOylation of JDP2 do not seem to interrupt the binding of JDP2-HDACs. However, more studies need to test whether PTMs of JDP2 disrupt the interaction of JDP2-HDACs. Overall, our results provide further evidence that PTMs are important for JDP2 activity. Since JDP2 has a significant role in biological and cellular processes, more studies are required to further validate the role of PTMs inn JDP2 functions.

In summary, we demonstrated that JDP2 through cAMP response elements activates *Mc2r* promoter activity and that PTMs (phosphorylation and SUMOylation) regulate JDP2 activity. However, more studies are needed to expand our understanding of how JDP2 coordinates with NR5A1 and FOXL2 to influence MC2R activity in adrenal glands and adrenocortical cancers. Collectively, our results not only extend the conclusion that JDP2 is involved in the steroidogenesis in adrenal glands through MC2R but also provide the novel mechanism for how PTMs regulate JDP2 activity.

## 4. Materials and Methods

### 4.1. Reagents

All cell culture reagents were purchased from Life Technologies (Carlsbad, CA, USA). Antibodies against JDP2, MC2R, and β-Actin (Santa Cruz Biotechnology Inc., Santa Cruz, CA, USA), SUMO1 and SUMO3 (Active motif, Carsbad, CA, USA), NR5A1 (Upstate Biochemistry Inc., Charlottsville, VA, USA), and FLAG (Sigma, St. Louis, MO, USA) were purchased commercially. Luciferase activity was measured using the Dual Luciferase Assay System (Promega, Madison, WI, USA). GST-JDP2 recombinant proteins were purchased from Abnova (Taipei, Taiwan).

### 4.2. DNA Constructs

Mouse HIS-FLAG-*Jdp2* cDNA was PCR-amplified and ligated into the HindIII and BamHI sites of pcDNA3^+^ to create pcDNA3-HIS-FLAG-*Jdp2* expression plasmid. T148A, T148D, and 1-100 Jdp2 expression plasmids were created by PCR-based mutagenesis (QuikChange Lightning site-directed mutagenesis kit, Agilent/Strategene, La Jolla, CA, USA). The murine *Mc2r* promoter (−2000-bp) and deletion constructs were previously generated in our laboratory, as described by Yang et al. [34]. The cAMP response element mutations (TGA or TCA were mutated to ACC) were created by PCR-based mutagenesis (QuikChange Lightning site-directed mutagenesis kit, Agilent/Strategene, La Jolla, CA, USA). Human *ATF3*-pGL2 promoter construct was kindly provided by Ami Aronheim (Technion-Israel Institute of Technology, Haifa, Israel.). All constructs were verified by nucleotide sequencing.

### 4.3. Cell Culture and Transfection

MCF7, HepG2, Ishikawa, Y1, and JEG3 cells were obtained from the American Type Culture Collection (Manassas, VA, USA). Cells were maintained in Dulbecco’s-modified eagle medium (DMEM) supplemented with 10% fetal bovine serum and antibiotics (GIBCO/Life Technologies, Grand Island, NY, USA) in humidified air containing 5% CO_2_ at 37 °C and cultured for less than six months. After incubation, the cells were transfected using Fugene HD Transfection Reagent (Roche, Madison, WI, USA) according to manufacturer’s instructions. Forty-eight hours after transfection, the cells were harvested and lysed. Luciferase activity was measured and normalized with Renilla activity. All experiments using transfection method were performed three times in triplicate.

### 4.4. RT-PCR and Real-Time ChIP

The animal study was approved by the University of Alabama at Birmingham Institutional Animal Care and Use Committee (Animal project Number: IACUC-20081). Adrenal glands were harvested from normal adult mice and frozen in a dry-ice ethanol bath and then stored at −80 °C until use. Total RNA from adrenal glands were extracted using TRIzol reagent and treated with DNase (Ambion, Austin, TX, USA) to remove genomic DNA. The RNA concentration was quantified by ultraviolet spectrometry. One microgram of total RNA was used to synthesize cDNA using the iScript kit (Bio-Rad) according to the manufacturer’s instructions. The final cDNA product was purified and eluted in Tris-EDTA buffer using QIAquick PCR purification kits (QIAGEN, Venlo, Netherlands) according to manufacturer’s instructions. Two primers (5′-CCTGGGCAGATCCCAGAC-3′ and 5′-CTTCACGGGTTGGGGCCTC-3′) were used to amplify 189-bp *Jdp2* fragments. Two primers (5′-CCTGAACAACCACAGCCTCGTAAAGGAC-3′ and 5′-CTGCATGCTCAGGGCCCGCACCTCCACC-3′) were used to amply 147-bp mouse *Nr5a1* fragment. Two primers (5′-CATCACCATCTTCCAGGAGCGA-3′ and 5′-GTCTTCTGGGTGGCAGTGATGG-3′) were used to amplify 341-bp mouse glyceraldehydes-3-phosphate dehydrogenase (*Gapdh*) fragments. For real-time ChIP, the extracted DNA fragments were quantified by real-time PCR using pairs of primers that covered the cAMP response region within the mouse *Mc2r* promoter (between −1320-bp and −720-bp). The primers used for PCR were −830RE: CATCCTTTTGTTGATAGTTT (forward) and GTTTTGATTGGATCTGGA (reverse), −1080RE: CTTTTGTACAGTGTCACTAT (forward) and GCACAGAGGTTATAAGCAT (reverse), and −1320RE: GCTGCTCACAGCTAGCTGGC (forward) and GATATGCATCCAACGGGAT (reverse).

### 4.5. Immunoblotting

Y1 or HepG2 cells (2 × 10^6^) were plated onto 100-mm cell culture dishes. Forty-eight hours after transient transfection, cells were harvested and lysed. Protein lysates were placed onto a rotator to rotate at 4 °C for 30 min, and protein concentrations of the high-speed supernatant were quantified using the BCA™ Protein Assay kit assay (Pierce/Thermo Scientific, Rockford, IL, USA). Immunoblotting was performed as previously described [26,30,31]. Equivalent quantities of protein (30–40 µg) were resolved on polyacrylamide-SDS gels, transferred to polyvinylidene difluoride (PVDF) membrane (Bio-Rad, Hercules, CA, USA), and immunoblotted with specific antibodies. The immune detection was done with the Supersignal West Dura Extended Duration Substrate kit (Pierce Chemical Co., Rockford, IL, USA). Protein band intensity was quantified by ImageJ program (bundled with 64-bit Java 1.6.0_24).

### 4.6. Cell-Free SUMOylation Assays

The cell-free SUMOylation assays were performed using a SUMOlink kit (Active motif, Carlsbad, CA, USA) according to the manufacturer’s instructions. Wild-type JDP2 full-length recombinant proteins with a GST tag (from Abnova, Taiwan) were incubated with wild-type SUMO1 (or wild-type SUMO3) or SUMO1 mutant (or SUMO3 mutant) along with SUMO E1 activating and SUMO E2 conjugating enzymes at 30 °C for 3 h. The SUMOylation reaction was terminated by adding an equal volume of 2× SDS–PAGE loading buffer. The samples were advanced to immunoblotting using anti-SUMO1 (or anti-SUMO3) or anti-JDP2 antibodies. The immune detection was carried out with the Supersignal West Dura Extended Duration Substrate kit (Pierce Chemical Co., Rockford, IL, USA). Protein band intensity was determined by ImageJ program.

### 4.7. Statistical Analysis

Statistical analyses were done by using the Student’s *t* test. When more than two groups were compared, a one-way ANOVA was performed. After the ANOVA analysis, the post hoc multiple comparisons were determined by using Tukey’s honest significant difference (HSD) test to determine the statistical difference between subgroups. For each test, *p* < 0.05 was considered to indicate significant difference.

## 5. Conclusions

In summary, this investigation demonstrated that JDP2, one of AP-1’s repressors, is a novel activator of *Mc2r* promoter, and that the post-translational modifications (phosphorylation and SUMOylation) play a critical role for JDP2’s transcriptional activity. Our study also adds a new layer of information to the previous understanding of how JDP2 functions in regulating adipocyte and skeletal muscle differentiation, interacting with and regulating progesterone receptor, controlling bone homeostasis and antibacterial immunity, and contributing to metastasis.

## Abbreviations

JDP2	Jun dimerization protein 2
MC2R	Melanocortin 2 receptor
SUMO	Small ubiquitin-like modifier
